# Topological confinement in an antisymmetric potential in bilayer graphene in the presence of a magnetic field

**DOI:** 10.1186/1556-276X-6-452

**Published:** 2011-07-14

**Authors:** Mohammad Zarenia, Joao Milton Pereira, François Maria Peeters, Gil de Aquino Farias

**Affiliations:** 1 Department of Physics, University of Antwerp, Groenenborgerlaan 171, B-2020 Antwerpen, Belgium; 2 Departamento de Física, Universidade Federal do Ceará, Fortaleza, Ceará, 60455-760, Brazil

## Abstract

We investigate the effect of an external magnetic field on the carrier states that are localized at a potential kink and a kink-antikink in bilayer graphene. These chiral states are localized at the interface between two potential regions with opposite signs.

PACS numbers: 71.10.Pm, 73.21.-b, 81.05.Uw

## Introduction

Carbon-based electronic structures have been the focus of intense research since the discovery of fullerenes and carbon nanotubes[1]. More recently, the production of atomic layers of hexagonal carbon (graphene) has renewed that interest, with the observation of striking mechanical and electronic properties, as well as ultrarelativistic-like phenomena in condensed matter systems[2-4]. In that context, bilayer graphene (BLG), which is a system with two coupled sheets of graphene, has been shown to have features that make it a possible substitute of silicon in microelectronic devices. The carrier dispersion of pristine BLG is gapless and approximately parabolic at two points in the Brillouin zone (K and K'). However, it has been found that the application of perpendicular electric fields produced by external gates deposited on the BLG surface can induce a gap in the spectrum. The electric field creates a charge imbalance between the layers which leads to a gap in the spectrum[5,6]. The tailoring of the gap by an external field may be particularly useful for the development of devices. It has been recently recognized that a tunable energy gap in BLG can allow the observation of new confined electronic states[7,8], which could be obtained by applying a spatially varying potential profile to create a position-dependent gap analogous to semiconductor heterojunctions.

**Figure 1:**
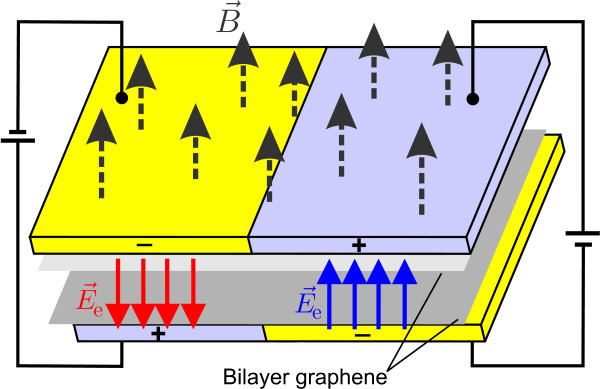
**(Color online) Schematic illustration of the bilayer graphene device for the creation of a kink potential**. Applied gated voltage to the upper and lower layers with opposite sign induce a spacial dependent electric field **E_e_**. An external magnetic field , is applied perpendicular to the bilayer graphene sheets.

An alternative way to create one dimensional localized states in BLG has recently been suggested by Martin *et al.*[9] and relies on the creation of a potential "kink" by an asymmetric potential profile (see Figure 1). It has been shown that localized chiral states arise at the location of the kink, with energies inside the energy gap. These states correspond to uni-directional motion of electrons which are analogous to the edge states in a quantum Hall system and show a valley-dependent propagation along the kink. From a practical standpoint, the kinks may be envisaged as configurable metallic nanowires embedded in a semiconductor medium. Moreover, the carrier states in this system are expected to be robust with regards to scattering and may display Luttinger liquid behavior[10]. Such kink potentials can be realized in e.g. p-n junctions. Recently the transport properties of p-n-p junctions in bilayer graphene were investigated experimentally in the presence of a perpendicular magnetic field[11].

An additional tool for the manipulation of charge states is the use of magnetic fields. The application of an external magnetic field perpendicular to the BLG sheet causes the appearance of Landau levels which can be significantly modified by the induced gap, leading to effect s such as the lifting of valley degeneracy caused by the breaking of the inversion symmetry due to the electrostatic bias[12,13]. The presence of a magnetic field in conjunction with electrostatic potential barriers in BLG has been shown to lead to a rich set of behaviors in which Landau quantization competes with the electrostatic confinement-induced quantization[14].

In the present work we investigate the properties of localized states in a kink potential profile under a perpendicular external magnetic field, both for the case of a single potential kink, as well as for a kink-antikink pair. One advantage of such a setup is the fact that in an experimental realization of this system the number of one-dimensional metallic channels and their subsequent magnetic response can be configurable, by controlling the gate voltages. As shown by our numerical results, the influence of the magnetic field can be strikingly distinct for single and double kinks.

## Model

We employ a reduced two-band continuum model to describe the BG sheet. In this model, the system is described by four sublattices in the upper (*A, B*) and lower (*A*' and *B*') layers[2]. The interlayer coupling is given by the hopping parameter *t *≈ 400 meV between sites *A *and B'. The Hamiltonian around the *K *valley of the first Brillouin zone can be written as(1)

where π = *v_F _*(*p_x _*+ *ip_y_*), *p_x, y _*= -*iħ∂*_*x,y *_+ *eA_x_*_,*y *_is the momentum operator in the presence of an external magnetic field with *A_x_*_,*y *_being the components of the vector potential **A**, *v_F _*= 10^6 ^m/s is the Fermi velocity, *U*(*x*) and -*U*(*x*) is the electrostatic potential applied to the upper and lower layers, respectively. The eigenstates of the Hamiltonian Eq. (1) are two-component spinors Ψ(*x, y*) = [*ψ*_a _(*x, y*), *ψ_b_*(*x, y*)]*^T^*, where *ψ_a_*_,*b *_are the envelope functions associated with the probability amplitudes at sublattices *A *and *B*' at the respective layers of the BLG sheet. We notice that [*H, p_y_*] = 0 and consequently the momentum along the *y *direction is a conserved quantity and therefore we can write,(2)

where, *k_y _*are the wave vector along the *y *direction. When applying a perpendicular magnetic field to the bilayer sheet we employ the Landau gauge for the vector potential **A **= (0, *B*_0_*x*, 0). The Hamiltonian (1) acts on the wave function of Eq. (2) which leads to the following coupled second-order differential equations,(3a)(3b)

where, in the above equations we used the dimensionless units *l *= *ħv_F_*/*t *= 1.6455 nm, *x*' = *x*/*l*, , ε *= E/t, u*(*x*') = *U*(*x*)/*t, β *= [*eB*_0_/*ħ*]*l*^2 ^(= 0.0041 for *B*_0 _= 1 *T*). The step-like kink (see Figure 1) is modeled by,(4)

where, *u_b _*is the maximum value of the gate voltage in dimensionless unit in each BLG layer. Here, *δ *denotes the width of the region in which the potential switches its sign in each layer. This parameter is determined by the distance between the gates used to create the energy gap. We solved numerically Eqs. (3) using the finite element technique to obtain the the spectrum as function of the magnetic field and the potential parameters.

## I. Numerical Results

**Figure 2:**
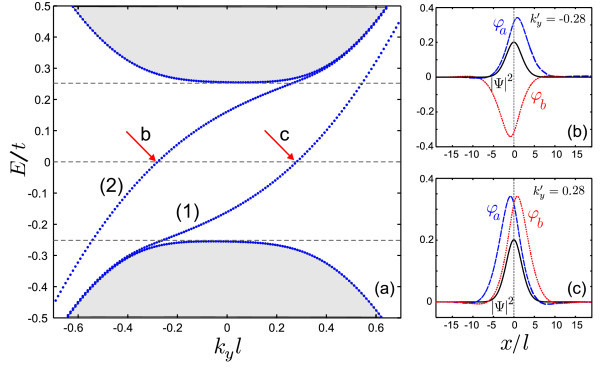
**Energy levels for a single kink profile on bilayer graphene in the absence of magnetic field with *u_b _*= 0.25 and *δ *= 1**. The right panels show the wave spinors and probability density corresponding to the states that are indicated by arrows in panel (a).

Figure 2(a) shows the spectrum for a potential kink as function of the wavevector along the kink for zero magnetic field. In this case, the potential kink is sharp, i.e. *δ *= 1 in Eq. (4). It is seen that the solutions of Eq. (3) for *B*_0 _= 0 are related by the transformations *ϕ_a _*→ - *ϕ_b_, ϕ_b _*→ *ϕ_a_, k_y _*→ - *k_y _*and *ε *→ -*ε*. The shaded region corresponds to the continuum of free states. The dashed horizontal lines correspond to *ε *= ±*u_b _*and *ε *= 0, with *u_b _*= 0.25. These results are found in the vicinity of a single valley (K) and show the unidirectional character of the propagation, in which only states with positive group velocity are obtained. Notice that the spectrum has the property . For localized states around the *K*' valley, we have *E_K_*_' _(*k_y_*) = - *E_K _*(*k_y_*). Panels (b) and (c) of Figure 2 present the spinor components and the probability density for the states indicated by the arrows in panel (a), corresponding to  (b) and  (c). These electron states are localized at the potential kink.

**Figure 3:**
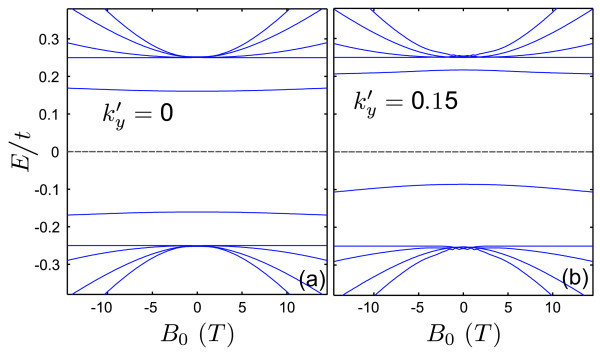
**Energy levels of a single kink profile in bilayer graphene as function of external magnetic field *B*_0 _**with **the same parameters as Fig. 2 for (a) ****and (b) **.

Figure 3 shows the dependence of the single kink energies on the external magnetic field for (a)  and (b) . The branches that appear for |*E*/*t*| > 0.25 correspond to Landau levels that arise from the continuum of free states. It is seen that the spectrum of confined states is very weakly influenced by the magnetic field. That is a consequence of the strong confinement of the states in the kink potential. In a semiclassical view, the movement of the carriers is constrained by the potential, which prevents the formation of cyclotron orbits.

**Figure 4:**
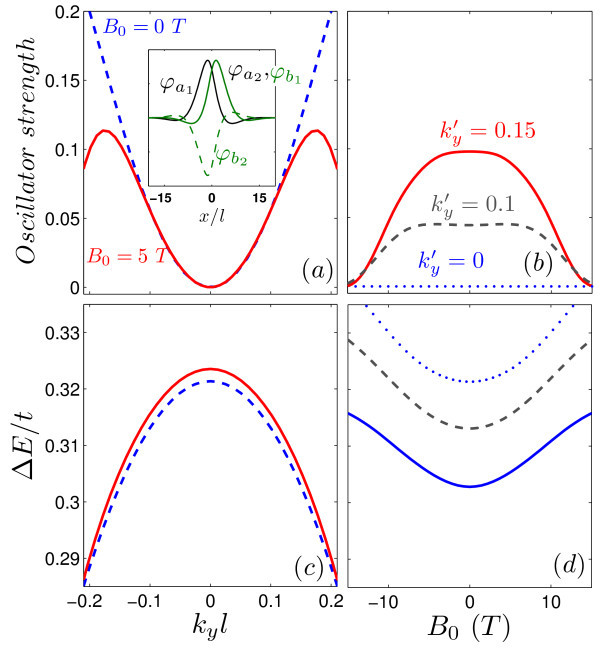
**(Color online) Oscillator strength for the transition between the topological states of the single kink profile (The states are labeled by (1), (2) in Fig. 2) and the corresponding transition energies Δ*E *as function of (a,c) the *y*-component of the wavelength  and (b,d) the external magnetic field *B*_0_**. The inset in (a) shows the wavespinors for *k_y_l *= 0.

We also calculate the oscillator strength for electric dipole transitions between the topological energy levels. The oscillator strength |<ψ*|*re^iθ^*| *ψ*>|^2 ^is given by(5)

where, *i *= *a, b*. Figure 4 shows the oscillator strength and the corresponding transition energy Δ*E *for the topological states of a single kink profile. The results are presented as function of  (panels (a,c)) and the external magnetic field (panels (b,d)). The topological states are indicated by (1), (2) in Figure 2(a). The  property of the topological levels leads to a symmetric behavior around  for the oscillator strength. The results in Figure 4(a) show a zero value for the oscillator strength at . As shown in the inset of Figure 4(a) the wavespinors for the first state  and the second one  at  are related as  and  which results | <*ψ*^†^| *x *|*ψ *> |^2 ^= 0 in Eq. (5). Panel 4(b) presents the oscillator strength as function of magnetic field for several values of . The presence of an external magnetic field decreases the oscillator strength at large momentum whereas the *B*_0 _= 0 result exhibits an increase in the oscillator strength (blue dashed curve in (a)). The reason is that a large magnetic field together with a large momentum weakly affects the topological states of the single kink profile (see Figure 3(b)). Note that the oscillator strength vs magnetic field is zero for  (dotted line in panel (b)).

**Figure 5:**
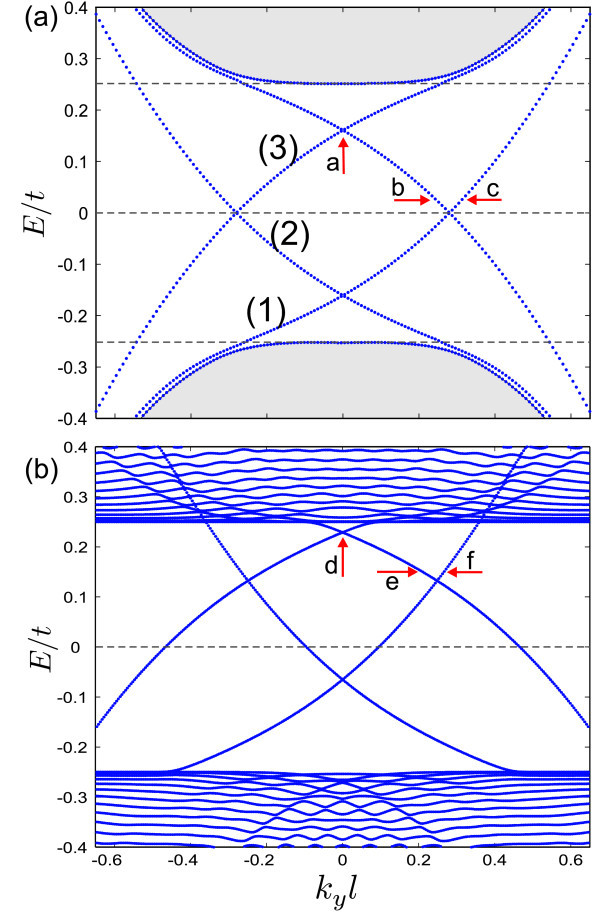
**Energy levels of a kink-antikink profile on bilayer graphene with *u_b _*= 0.25 and *δ *= 1 for (a) *B*_0 _= 0 *T *and (b) *B*_0 _= 3 *T***. The kinks are located at *x*' = ±15 (or *x *≈ ± 25 *nm *in real units).

**Figure 6:**
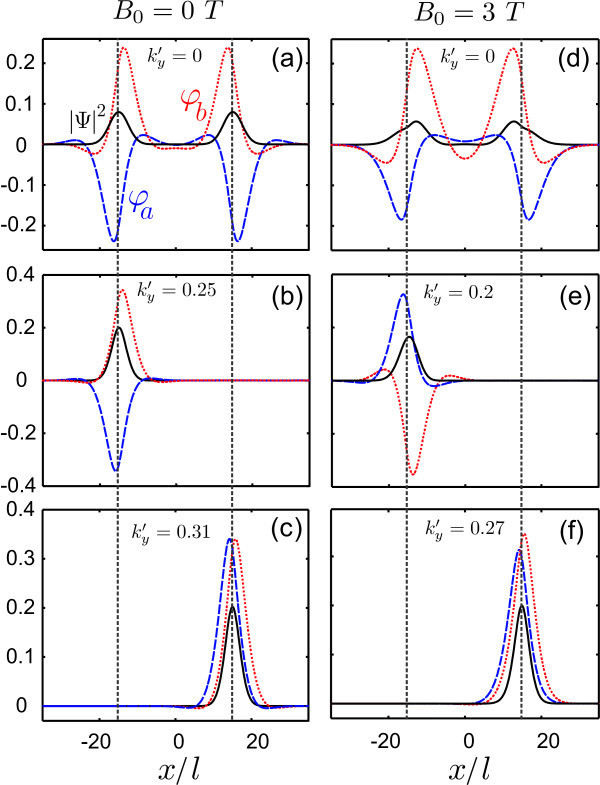
**Wave spinors, *φ_a_*, *φ_b _*and the corresponding probability density for the points in the energy spectrum which are indicated in Fig. 5 by arrows**.

Next we considered a potential profile with a kink-antikink. Figure 5 shows the spectrum of localized states for *B*_0 _= 0 (a) and *B*_0 _= 3 T (b). The results show a shift of the four mid-gap energy branches as the magnetic field increases. In addition, the continuum of free states at zero magnetic field is replaced by a set of Landau levels for *ε *>*u_b_*. The spinor components and probability densities associated with the points indicated by arrows in Figure 5(a) and Figure 5(b) are shown in Figure 6. In Figure 6(a) the wavefunction shows the overlap between states localized in both the kink and antikink, for zero magnetic field. With increasing wavevector, the states become strongly localized in either the kink (b) or antikink (c). Panels (d) to (f) show the wavefunctions for non-zero magnetic field. The states at , (panel (d)) show a shift of the probability density towards the central region of the potential. That is caused by the additional confinement brought about by the magnetic field. However, for a larger value of the wavevector, the wavefunctions are only weakly affected by the field, due to the strong localization of the states.

**Figure 7:**
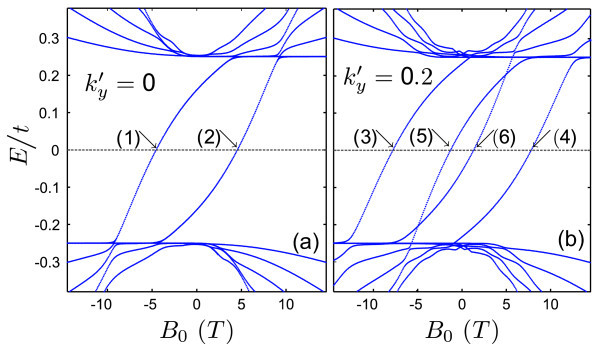
**Energy levels of a kink-antikink profile in bilayer graphene as function of external magnetic field *B*_0 _for (a) ****and (b) **. The other parameters are the same as Fig. 5.

Figure 7 displays the energy levels of a kink-antikink potential as function of an external magnetic field for (a)  and (b) . For the kink-antikink case, the overlap between the states associated with each confinement region allows the formation of Landau orbits. Therefore, in contrast to the single kink profile, the proximity of an antikink induces a strong dependence of the states on the external field.

The localization of the states is reflected in the position dependence of the current. The current in the *y*-direction is obtained using(6)

where . we rewrite Eq. (6) in the following form(7)

**Figure 8:**
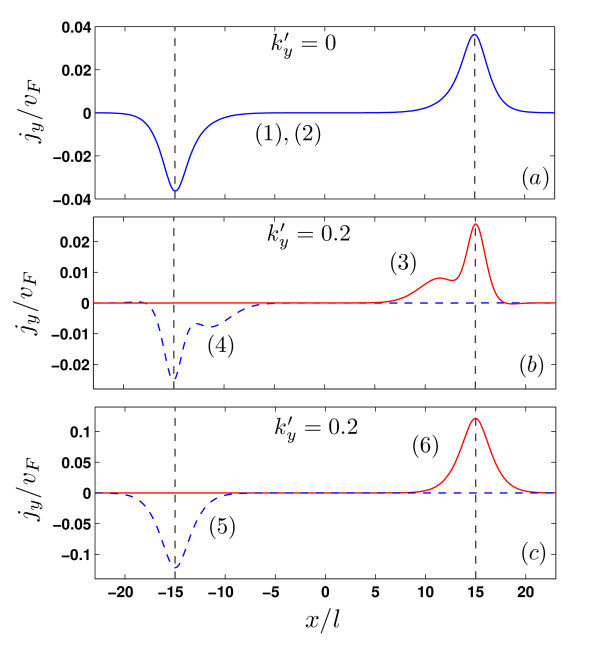
***y *component of the Persistent current in bilayer graphene as function of *x *direction for the values of magnetic field where *E *= *E_F _*which are indicated by (1), (2), ... in Fig. 7(a),(b)**.

The x-component of the current vanishes for the confined states. It should be noticed that a non-zero current can be found for *E *= 0, as can be deduced from the dispersion relations. Figure 8 shows plots of the *y*-component of the current density as function of *x *for the states labelled (1) to (6) in panels (a) and (b) of Figure 7. For  the results presented in Figure 8(a) show a persistent current carried by each kink region, irrespective of the direction of *B*_0_, as exemplified by the states (1) and (2) which correspond to opposite directions of magnetic field. For non-zero wave vectors, however, as shown in panels (b) and (c), the current is strongly localized around either potential kink. In Figure 8(b), the density current curve shows an additional peak caused by a stronger magnetic field (*B*_0 _≈ 10 *T *).

**Figure 9:**
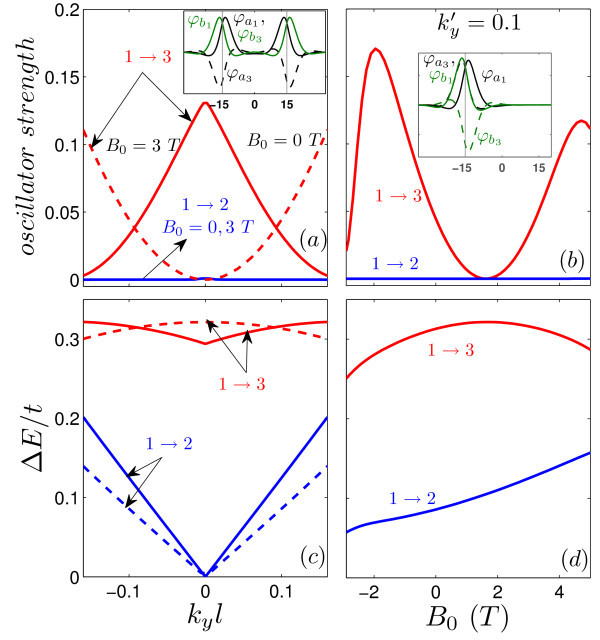
**(Color online) (a,b) Oscillator strength and (c,d) the corresponding transition energies **Δ*E ***for the 1 → 2 (blue curves) and 1 → 3 (red curves) transitions between the intragap energy states of the kink-antink profile as function of (a,c) ****and (b,d) the external magnetic field *B*_0 _(the energy levels are labeled by (1), (2), (3) in Fig. 5(a))**. Dashed curves and solid curves in panels (a,c) display the results respectively for a zero and non-zero magnetic field. The insets in panels (a),(b) show the wavespinors of the levels (1) and (3) corresponding to the points with zero oscillator strength.

Figure 9 displays the oscillator strength and the corresponding transition energy for the mid-gap levels of the kink-antikink potentials as function of (a,c)  and (b,d) external magnetic field *B*_0 _(the energy branches are labeled by (1), (2), (3) in Figure 5(a)). The wavefunction for the energies corresponding to the kink states (1), (3) are localized around *x' *= *d *whereas the antikink energy levels confine the carriers around *x *= - *d *and consequently the oscillator strength by the transition between the kink and the antikink states (e.g. 1 → 2) is zero in the absence or either presence of magnetic field (blue solid curves in panels (a,b)). The inset of panel (a) indicates that the wavespinors satisfy the  and  relations at  and *B*_0 _= 0 which leads to a zero oscillator strength for the 1 → 3 transition. In contrast to the single kink profile the shift in the intragap energies of the kink-antikink potential leads to a non-zero value for the oscillator strength at  (red solid curve in (a)). The oscillator strength as function of the external magnetic field is shown in panel (b) for . The inset in panel (b) shows the wavefunction of the states (1) and (3) at *B*_0 _≈ 1.6 *T *where, the same relations as for the single kink potential between the wavespinors ( and ) leads to a zero value for the oscillator strength.

## Conclusions

We obtained the spectrum of electronic bound states that are localized at potential kinks in bilayer graphene, which can be created by antisymmetric gate potentials. For a single potential kink, the bound states are only weakly influenced by an external magnetic field, due to their one-dimensional character, caused by the strong confinement along the direction of the potential kink interface. For a kink-antikink pair, however, the numerical results show a significant shift of the carrier dispersion, which arises due to the coupling of the states localized at either potential interface. Therefore, such configurable kink potentials in bilayer graphene permits the tailoring of the low-dimensional carrier dynamics as well as its magnetic field response by means of gate voltages.

## Competing interests

The authors declare that they have no competing interests.

## Authors' contributions

MZ carried out the numerical results JMP Jr and FMP were involved in the conception of the study and performed the sequence alignment and drafted the manuscript. GAF contributed in analysis of the numerical results. All authors read and approved the final manuscript.
